# HLA-A*0201-restricted CTL epitope of a novel osteosarcoma antigen, papillomavirus binding factor

**DOI:** 10.1186/1479-5876-7-44

**Published:** 2009-06-12

**Authors:** Tomohide Tsukahara, Satoshi Kawaguchi, Toshihiko Torigoe, Akari Takahashi, Masaki Murase, Masanobu Kano, Takuro Wada, Mitsunori Kaya, Satoshi Nagoya, Toshihiko Yamashita, Noriyuki Sato

**Affiliations:** 1 Department of Orthopaedic Surgery, Sapporo Medical University School of Medicine, South-1, West-16, Chuo-ku, Sapporo, 060-8543, Japan; 2 Department of Pathology, Sapporo Medical University School of Medicine, South-1, West-17, Chuo-ku, Sapporo, 060-8556, Japan

## Abstract

**Background:**

To develop peptide-based immunotherapy for osteosarcoma, we previously identified papillomavirus binding factor (PBF) as a CTL-defined osteosarcoma antigen in the context of HLA-B55. However, clinical application of PBF-based immunotherapy requires identification of naturally presented CTL epitopes in osteosarcoma cells in the context of more common HLA molecules such as HLA-A2.

**Methods:**

Ten peptides with the HLA-A*0201 binding motif were synthesized from the amino acid sequence of PBF according to the BIMAS score and screened with an HLA class I stabilization assay. The frequency of CTLs recognizing the selected PBF-derived peptide was determined in peripheral blood of five HLA-A*0201^+ ^patients with osteosarcoma using limiting dilution (LD)/mixed lymphocyte peptide culture (MLPC) followed by tetramer-based frequency analysis. Attempts were made to establish PBF-specific CTL clones from the tetramer-positive CTL pool by a combination of limiting dilution and single-cell sorting. The cytotoxicity of CTLs was assessed by ^51^Cr release assay.

**Results:**

Peptide PBF A2.2 showed the highest affinity to HLA-A*0201. CD8+ T cells reacting with the PBF A2.2 peptide were detected in three of five patients at frequencies from 2 × 10^-7 ^to 5 × 10^-6^. A tetramer-positive PBF A2.2-specific CTL line, 5A9, specifically lysed allogeneic osteosarcoma cell lines that expressed both PBF and either HLA-A*0201 or HLA-A*0206, autologous tumor cells, and T2 pulsed with PBF A2.2. Five of 12 tetramer-positive CTL clones also lysed allogeneic osteosarcoma cell lines expressing both PBF and either HLA-A*0201 or HLA-A*0206 and T2 pulsed with PBF A2.2.

**Conclusion:**

These findings indicate that PBF A2.2 serves as a CTL epitope on osteosarcoma cells in the context of HLA-A*0201, and potentially, HLA-A*0206. This extends the availability of PBF-derived therapeutic peptide vaccines for patients with osteosarcoma.

## Background

Osteosarcoma is the most common primary malignant tumor of bone. The survival rate of patients with osteosarcoma was under 20% before 1970. The introduction of neoadjuvant chemotherapy, establishment of guidelines for adequate surgical margins, and development of post-excision reconstruction raised the five-year survival rate to 60–70% [1,2]. These advances overshadowed the pioneering adjuvant immunotherapy trials using autologous tumor vaccines for patients with osteosarcoma, despite their having some therapeutic efficacy [3-5]. However, the survival rate of patients with osteosarcoma has reached a plateau in the last decade [6,7], which has reignited interest in immunotherapeutic approaches [8-10].

We previously identified papillomavirus-binding factor (PBF) as a novel osteosarcoma antigen, using an osteosarcoma cell line and an autologous CTL (cytotoxic T lymphocyte) clone restricted by HLA-B*5502 [11,12]. PBF is a DNA-binding transcription factor and a regulator of apoptosis [13-15]. PBF protein is expressed in 92% of osteosarcomas. Moreover, PBF-positive sarcomas have a significantly worse prognosis than PBF-negative sarcomas [16,17]. Development of PBF-based immunotherapy requires identification of naturally presented CTL epitopes in osteosarcoma cells in the context of common HLA molecules such as HLA-A2 and HLA-A24. The present study was designed to determine HLA-A*0201-restricted CTL epitopes from PBF.

## Methods

This study was approved under institutional guidelines for the use of human subjects in research. The patients and their families as well as healthy donors gave informed consent for the use of blood samples and tissue specimens in our research.

### Cells

The osteosarcoma cell lines OS2000 and KIKU were established in our laboratory [11,18]. The osteosarcoma cell lines U2OS, Saos-2 and HOS, human lymphoblastoid cell line T2, and erythroleukemia cell line K562 were purchased from ATCC (Manassas, VA). OS2000, KIKI, U2OS, Saos-2, HOS and K562 are PBF-positive [12]. U2OS, Saos-2, and T2 are HLA-A*0201 positive. The HLA genotypes of the osteosarcoma cell lines were as follows: OS2000, A*2402, B*5502, B*4002, Cw*0102; U2OS, A*0201, A*3201, B*4402, Cw*0501, Cw*0704; Saos-2, A*0201, A*2402, B*1302, B*4402, Cw*0602, Cw*0704; HOS, A*0211, B*5201, Cw*1202; KIKU, A*0206, A*2402, B*4006, B*5201, Cw*0802 and Cw*1202. Epstein-Barr virus-transformed B cell line NS-EBV-B was established from a healthy donor in our laboratory. Another Epstein-Barr virus-transformed B cell line, LCL-OS2000, was established from a patient with osteosarcoma [11].

Autologous tumor cells were developed from fresh frozen biopsy specimens of osteosarcoma. The specimens were thawed in Iscove's modified Dulbecco's modified Eagle's medium containing 10% FCS at room temperature, minced into small pieces and filtrated with a 70 μm Cell Strainer (BD Biosciences, Bedford, MA). The cells were used immediately for cytotoxicity assay.

### Design and synthesis of PBF-derived peptides

Based on the entire amino acid sequence of PBF, peptides with the ability to bind to HLA-A*0201 class I molecules were searched for through the World Wide Web site Bioinformatics and Molecular Analysis Section (BIMAS) HLA Peptide Binding Predictions [19]. Based on the binding scores, ten peptides were selected and synthesized [see Additional file 1].

### HLA class I stabilization assay

The affinity of peptides for HLA-A*0201 molecules was evaluated by T2 cell surface HLA class-I stabilization assay as described previously [20,21]. An HLA-A*0201-binding influenza matrix protein-derived peptide (Inf-MP A2; GILGFVFTL) [22] was used for positive control. Mouse H-2Kb-restricted peptide VSV8 (RGYVYQGL) [23] was used for negative control. Assays were performed in triplicate. The affinity of each peptide for HLA-A*0201 molecules was evaluated by the percent mean fluorescence intensity (%MFI) increase of the HLA-A*0201 molecules detected by staining with an anti-HLA-A2 monoclonal antibody (BB7.2, purchased from ATCC) using the following calculation. %MFI increase: [(MFI with the given peptide – MFI without peptide)/(MFI without peptide)] × 100.

### Limiting dilution/mixed lymphocyte peptide culture

Prior to frequency analysis and cytotoxicity assays, PBMC of patients were subjected to mixed lymphocyte peptide culture under limiting dilution conditions (LD/MLPC) according to the method described by Karanikas et al. [24] with some modifications [17]. LD/MLPC aims to seed at most one CTL precursor cell per well and induces proliferation of the precursor cell by subsequent mixed lymphocyte peptide culture. For this purpose, the appropriate number of PBMC and CD8^+ ^cells per well is considered to be 1 × 10^5^–2 × 10^5 ^[17,24].

PBMCs were used as a source of responder cells in the initial five subjects (Patients 1 and 2 and three healthy donors) and CD8^+ ^cells were used in the following three patients (Patients 3–5) [see Additional file 2].

PBMC obtained from peripheral blood samples (50 ml) of Patients 1 and 2 and three healthy donors were suspended in AIM-V (Invitrogen Corp., Carlsbad, CA) supplemented with 1% human serum (HS). These cells were incubated for 60 min at room temperature with peptide PBF A2.2 (50 μg/ml). Peptide-pulsed PBMC were seeded at 2 × 10^5 ^cells/200 μl/well into round-bottom 96-microwell plates in AIM-V with 10%HS, IL-2 (20 U/ml; a kind gift from Takeda Chemical Industries, Ltd., Osaka Japan) and IL-7 (10 ng/ml; R&D Systems, Minneapolis, Minnesota, USA), and incubated. On day 7, half of the medium was replaced with fresh AIM-V containing IL-2, IL-7 and the same peptides. The cell cultures were maintained by adding fresh AIM-V containing IL-2. On days 14–21, they were subjected to tetramer-based frequency analysis.

PBMC obtained from Patients 3–5 were separated into CD8^+ ^cells and CD8^- ^cells using magnetic anti-CD8 microbeads (Miltenyi Biotec, Gladbach, Germany). CD8^- ^cells were pulsed with the PBF A2.2 peptide for 60 min. Half of the CD8^- ^cells were cryopreserved at -80°C for the second stimulation. CD8^+ ^cells (1.0–2.1 × 10^5^/well) and irradiated PBF A2.2 peptide-pulsed CD8^- ^cells (1–5 × 10^5^/well) were cocultured in 48-well cell culture plates in 500 μl of AIM-V with 10%HS, IL-2 and IL-7. On day 7, the second stimulation was performed by adding irradiated peptide-pulsed CD8^- ^cells to each culture well in 500 μl of AIM-V with 10%HS, IL-2 and IL-7. On days 13–23, they were subjected to tetramer-based frequency analysis.

### Tetramer-based frequency analysis

An FITC-conjugated HLA-A*0201/HIV tetramer (here termed the control tetramer) and a PE-conjugated HLA-A*0201/PBF A2.2 tetramer (A2/PBF A2.2 tetramer) were constructed by Medical & Biological Laboratories Co., Ltd. (Tokyo, Japan). PBMCs from patients were stimulated with the PBF A2.2 peptide by using the LD/MLPC procedure as described above. From each microwell containing 200 μl of the microculture pool, 100 μl was transferred to a V-bottom microwell and washed. To the spin-down pellets, the control tetramer and A2/PBF A2.2 tetramer (10 nM in 25 μl of PBS) were added in combination and incubated for 15 min at room temperature. Then a PE-Cy5-conjugated anti-CD8 antibody (eBioscience, San Diego, California, USA) was added (dilution of 1:30 in 25 μl of PBS containing the control tetramer and A2/PBF A2.2 tetramer) and incubated for another 15 min. The cells were washed in PBS twice, fixed with 0.5% formaldehyde, and analyzed by flow cytometry using FACScan and CellQuest software (Becton Dickinson, San Jose, California, USA). CD8^+ ^living cells were gated and the cells labeled with the A2/PBF A2.2 tetramer were referred to as tetramer-positive cells. Tetramer-positive cells in each well are theoretically derived from a single CTL precursor, regardless of the number (percentage) of tetramer-positive cells. Accordingly, the number of tetramer-positive wells represents the number of CTL precursors. The frequency of anti-PBF A2.2 CTLs was evaluated using the following calculation: (number of tetramer-positive wells)/[(total number of tested wells) × (initial number of CD8+ cells per well)].

### Development of CTL line and CTL clones

Attempts to establish CTL clones were made by a limiting dilution procedure and subsequent single-cell sorting procedures.

In the limiting dilution procedure, cells from a tetramer-positive T cell pool derived from Patient 4 were replated into a 96-well round-bottom microplate at one cell per well. In each well, a T cell was cocultured with irradiated A*0201^+ ^NS-EBV-B cells (2 × 10^4^) pulsed with the PBF A2.2 peptide and irradiated allogeneic PBMCs (8 × 10^4^) in 200 μl of AIM-V containing 10%HS, IL-2 (200 U/ml) and IL-7 (10 ng/ml). On days 7, 14 and 21, the stimulation was repeated by adding irradiated peptide-pulsed NS-EBV cells (1 × 10^4^), LCL-OS2000 cells (1 × 10^4^), and allogeneic PBMCs (8 × 10^4^) to each culture well in 100 μl of freshly replaced AIM-V with 10%HS, IL-2 and IL-7. On day 35, tetramer staining of all wells was performed. The tetramer-positive population was selected and further expanded. These cells were seeded at 2 × 10^3 ^per well with irradiated allogeneic PBMCs (1 × 10^5^) in 100 μl of AIM-V containing 10% HS, IL-2 (200 U/ml) and phytohemagglutinin-P (PHA; 7.5 μg/ml, Wako Chemicals, Osaka, Japan) in a total of 192 wells of 96-well round-bottom microplates. On day 7, 100 μl of AIM-V containing 10% HS and IL-2 was added. On day 14, all proliferated cells were collected, washed and replaced with fresh AIM-V containing 10% HS and IL-2, followed by maintenance in a 48-well microplate at 0.5–1 × 10^6 ^cells per well. The established oligoclonal cell line was designated CTL 5A9.

Subsequently, a frozen stock of the oligoclonal CTL 5A9 was reactivated and subjected to single-cell sorting. In the reactivation procedure, thawed CTL 5A9 cells were cultured with allogeneic PBMCs in AIM-V containing 10% HS, IL-2 (200 U/ml) and PHA (7.5 μg/ml) for 27 days. The reactivated CTL 5A9 cells were stained by the A2/PBF A2.2 tetramer and the control tetramer. The tetramer-positive cells (0.82%) were sorted at one cell per well using FACS Aria II (Becton Dickinson) with allogeneic PBMCs (1 × 10^5^) to each culture well in 200 μl of AIM-V with 10% HS, IL-2 (200 U/ml) and PHA (7.5 ug/ml) in a total of 384 wells of 96-well microplates. On days 7, 10 and 14, half of each medium was replaced with fresh medium without PHA. On days 20–34, tetramer staining was performed. Single-cell sorting was repeated until tetramer staining showed single clone populations.

### Cytotoxicity assay

CTL-mediated cytolytic activity was measured by a 6 h-^51^Cr-release assay [25]. Osteosarcoma cell lines (U2OS, OS2000, Saos-2, KIKU and HOS), K562, T2, and autologous osteosarcoma cells obtained from Patient 4 were used as target cells. T2 cells were treated with or without peptides at the indicated concentrations for 1 h at room temperature after ^51^Cr-labeling. An HIV peptide (SLYNTVATL)[26] was used as a negative control peptide. Target cells were labeled with 100 μCi of ^51^Cr for 1 h at 37°C. The labeled target cells were suspended in RPMI without serum and seeded to microwells (2–5 × 10^3 ^cells/well).

CTL 5A9 and CTL clones were used as the effector cells. The effector cells were transferred to V-bottom microwells, suspended in AIM-V and mixed with the labeled target cells. After a 6 h incubation period at 37°C, the ^51^Cr level in the culture supernatant was measured using an automated gamma counter. The percentage of specific cytotoxicity was calculated as follows: the percentage of specific ^51^Cr release = 100 × (experimental release – spontaneous release)/(maximum release – spontaneous release).

## Results

### Affinity of PBF-derived synthetic peptides to HLA-A*0201 molecules

**Figure 1:**
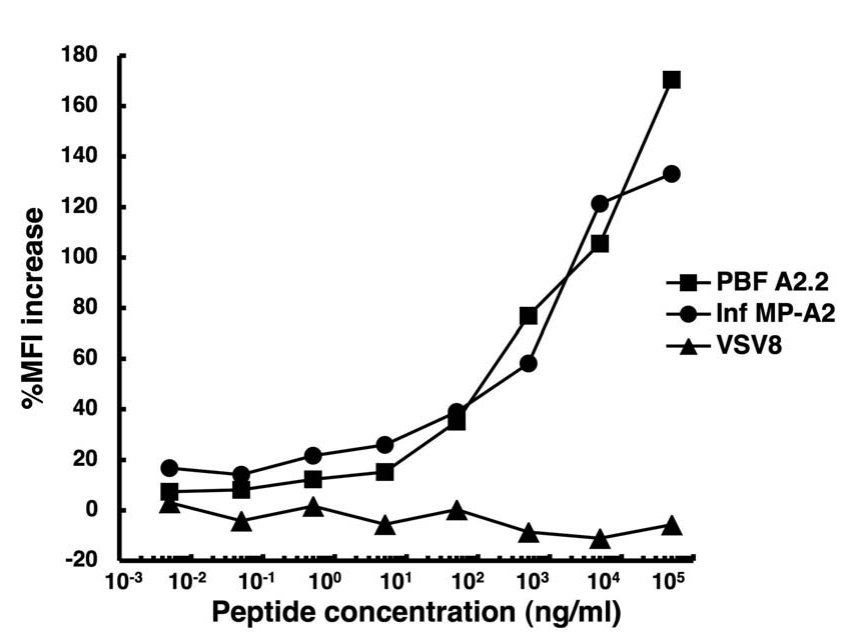
Binding affinity of PBF A2.2 peptide to HLA-A*0201 molecules. The affinities of three peptides, PBF A2.2, Inf MP-A2 and VSV8, were determined by HLA class I stabilization assay at the indicated concentrations.

To determine HLA-A*0201-restricted epitopes of PBF, we synthesized 10 peptides from the amino acid sequence of PBF in accordance with the BIMAS scores for HLA-A*0201 affinity [see Additional file 1]. Subsequently we evaluated the affinity of these peptides to HLA-A*0201 molecules by HLA class I-stabilization assay [see Additional file 1]. Peptide PBF A2.2 showed the highest %MFI increase among the peptides. Peptide titration experiments (Fig. 1) revealed dose-dependent increases of %MFI by PBF A2.2 and the positive control Inf-MP A2 peptide, but not the VSV8 negative control peptide.

### Frequency of anti-PBF A2.2-specific T cells in HLA-A*0201+ patients with osteosarcoma and healthy donors

**Figure 2:**
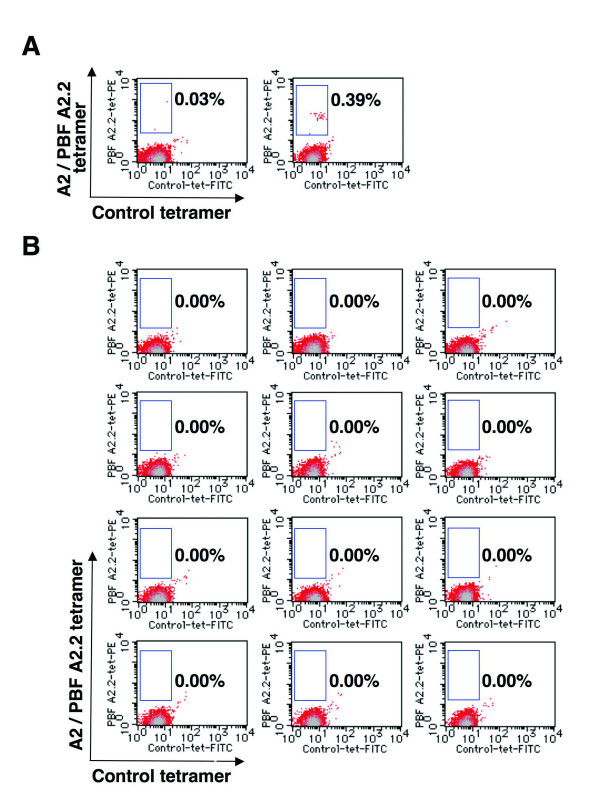
**Tetramer-based detection of PBF A2.2-specific T cells**. CD8+ T cells (5.4 × 10^6^) collected from Patient 4 were seeded into 36 wells at the concentration of 1.5 × 10^5 ^per well and cultured with peptide PBF A2.2 and cytokines. On day 21, tetramer analysis was performed. This analysis showed that 2 of 36 wells were positive, containing 0.03% and 0.39% tetramer-positive cells, respectively (A). The remaining 34 wells were negative with 0.00% reactivity. Here, 12 of 34 tetramer-negative wells are shown (B). Each of the 2 positive wells contained at least 1 CTL precursor, indicating that there were at least 2 CTL precursors in a total of 5.4 × 10^6 ^CD8+ cells. The frequency was calculated as 2/5.4 × 10^6 ^= 3.7 × 10^-7^.

We then examined the frequency of peripheral CD8^+ ^T-lymphocytes that recognized the PBF A2.2 peptide in five HLA-A*0201^+ ^patients with PBF-positive osteosarcoma by LD/MLPC/tetramer analysis. A2/PBF A2.2 tetramer-positive T cells were detected in three of the five patients [see Additional file 2]. Fig. 2 presents the results of flow cytometric analysis of Patient 4, showing two tetramer-positive wells and 12 of 34 tetramer-negative wells. This indicated the presence of at least two CTL precursor cells (PBF A2.2-specific CD8^+ ^T cells) in 5.4 × 10^6 ^CD8+ T cells examined. The frequencies of the PBF A2.2-specific CD8^+ ^T cells ranged from 2 × 10^-7 ^to 5 × 10^-6 ^(2 × 10^-6 ^on average) in three tetramer-positive patients. In the three healthy donors, the PBF A2.2-specific CD8^+ ^T cells ranged from 1 × 10^-7 ^to 3 × 10^-7 ^(2 × 10^-7 ^on average).

### Establishment of A2/PBF A2.2 tetramer-positive CTL oligoclonal line and CTL clones

**Figure 3:**
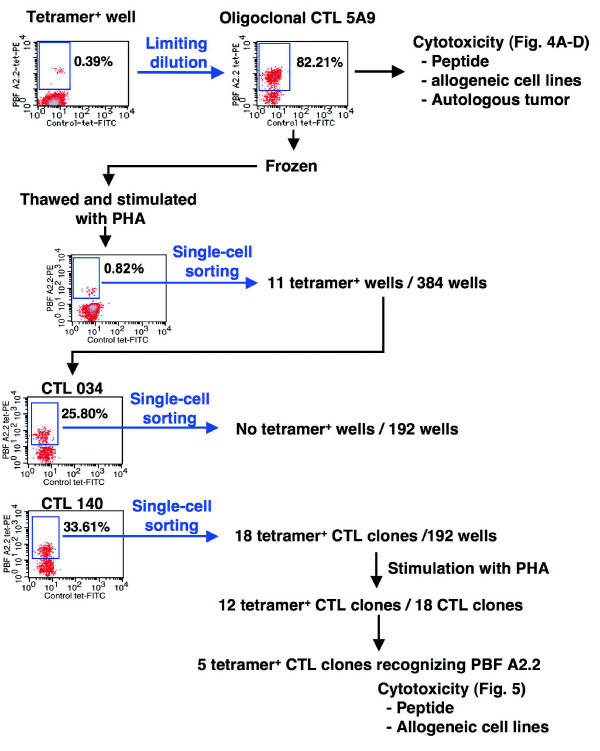
**Establishment of PBF A2.2-specific CTL line and CTL clones**.

Attempts to establish CTL clones were made by a combination of limiting dilution and repeated single-cell sorting. Limiting dilution of one of the tetramer-positive T cell pools from Patient 4 yielded a cell population (designated CTL 5A9) with more than 80% tetramer-positive CD8^+ ^cells (Fig. 3). RT-PCR analysis of TCR expression in CTL 5A9 revealed four V alpha mRNAs (V alpha 3, 5, 8 and 12) and clonal V beta mRNA (V beta 13.1) (data not shown), indicating the oligoclonal nature of CTL 5A9.

We then performed single cell sorting of CTL 5A9 (Fig. 3). The first single-cell sorting resulted in 11 tetramer-positive oligoclonal populations. Two of these 11 oligoclones were subsequently subjected to the second single cell sorting. From one oligoclone (clone 140), 12 single clones were established. Of these, five clones (1B1, 1D7, 1E1, 1F4 and 1F7) showed cytotoxic activity to PBF A2.2-pulsed T2 cells.

### Cytotoxicity of A2/PBF A2.2 tetramer-positive CTL oligoclonal line and CTL clones

**Figure 4:**
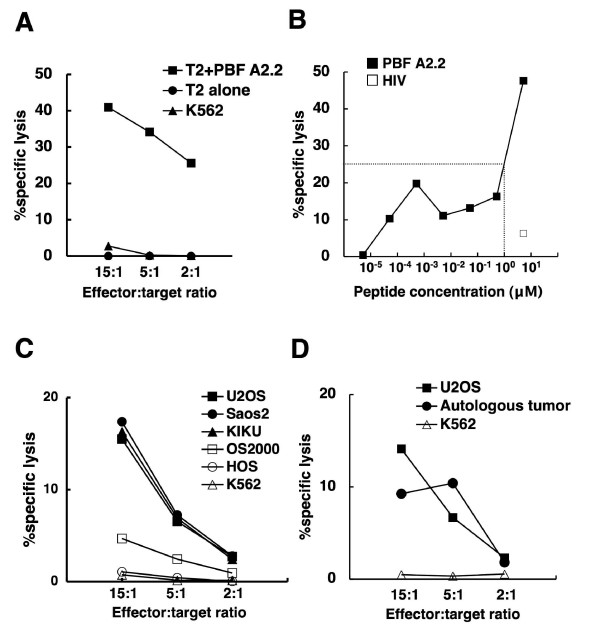
**Cytotoxic activity of A2/PBF A2.2 tetramer-positive CTL line 5A9**. **A**. The peptide-specific cytotoxicity of CTL 5A9 was determined using T2 and K562 cells in a 6 h standard ^51^Cr release assay. T2 cells were pulsed with 50 μg/ml peptide PBF A2.2 or medium for 1 h at room temperature after labeling with ^51^Cr. CTL 5A9 lysed PBF A2.2 peptide-pulsed T2 cells in an effector:target ratio-dependent manner, but not K562 or T2 cells without peptide pulsation. **B**. T2 cells were incubated with various concentrations of the PBF A2.2 peptide and 5 μM HIV control peptide. The cytotoxicity of CTL 5A9 against peptide-pulsed T2 cells was determined at an effector to target ratio of 30:1. Dotted lines indicate half maximum lysis. **C**. The cytotoxicity of CTL 5A9 against allogeneic osteosarcoma cell lines U2OS, Saos-2, KIKU, OS2000 and HOS. All cell lines express PBF. U2OS and Saos-2 are HLA-A*0201-positive. KIKU is HLA-A*0201-negative, HLA-A*0206-positive. OS2000 and HOS are HLA-A*0201-negative. **D**. Autologous tumor cells were derived from fresh-frozen biopsy specimens of Patient 4, from whom CTL 5A9 was also developed. U2OS and K562 were used as positive control target cells and natural killer target cells, respectively.

Finally we examined the cytotoxic properties of the oligoclonal line, 5A9, and five CTL clones. As shown in Fig. 4A, CTL 5A9 lysed PBF A2.2 peptide-pulsed T2 cells in an effector:target ratio-dependent manner. In contrast, such cytotoxic activity of CTL 5A9 was not seen against T2 cells without peptide pulsation or K562 cells. Cytotoxic activity of CTL 5A9 against PBF A2.2-pulsed T2 cells was dependent on the concentration of the PBF A2.2 peptide (Fig. 4B). Given the oligoclonal nature of CTL 5A9, we also examined the peptide-specific cytotoxicity of their tetramer-negative subpopulation. The tetramer-negative 5A9 subpopulation did not react against T2 cells, PBF A2.2 peptide-pulsed T2 cells, or K562 cells (data not shown).

Fig. 4C shows the cytotoxic activity of CTL 5A9 against osteosarcoma cells. CTL 5A9 exhibited cytotoxicity against U2OS (PBF-positive, HLA-A*0201-positive), Saos-2 (PBF-positive, HLA-A*0201-positive), and KIKU (PBF-positive, HLA-A*0201-negative, HLA-A*0206-positive) in an effector:target ratio-dependent manner. In contrast, CTL 5A9 showed marginal cytotoxicity against OS2000 (PBF-positive, HLA-A*0201-negative), and undetectable levels of cytotoxicity against HOS (PBF-positive, HLA-A*0201-negative) and K562 cells (PBF-positive, HLA-null). To assess the possibility of an allogeneic reaction for the cytotoxicity of CTL 5A9, we developed autologous tumor cells from fresh-frozen biopsy specimens of Patient 4 and used them as target cells. As shown in Fig. 4D, CTL 5A9 also lysed autologous tumor cells as well as the positive control, U2OS cells, but not K562 cells.

**Figure 5:**
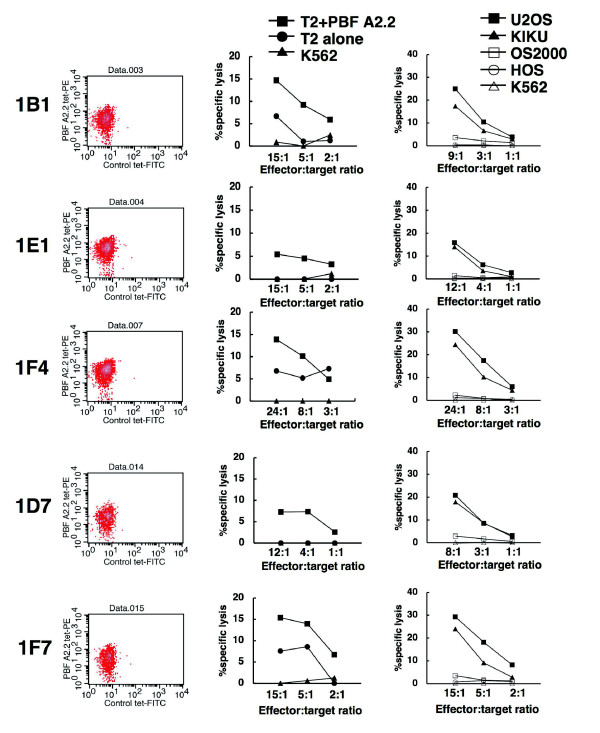
**Cytotoxic activity of CTL clones derived from CTL 5A9**. Five CTL clones were established from CTL 5A9. Left panels indicate tetramer staining of CTL clones. CD8^+ ^cells were gated. X-axis and Y-axis indicate the fluorescence intensity of control tetramer-FITC and A2/PBF A2.2 tetramer-PE, respectively. Middle panels indicate CTL-mediated cytotoxicity against T2 cells with or without PBF A2.2 peptide-pulsation. Right panels indicate CTL-mediated cytotoxicity against allogeneic osteosarcoma cell lines.

To further determine the specificity of A2/PBF A2.2 tetramer-positive CTLs against osteosarcoma cells in the context of HLA-A2, we analyzed the cytotoxicity of five CTL clones derived from CTL 5A9 (Fig. 5). All five CTL clones lysed PBF A2.2 peptide-pulsed T2 cells and osteosarcoma cell lines U2OS and KIKU. In contrast, none of five clones recognized OS2000, HOS or K562.

## Discussion

In the present study, we examined the immunogenicity of an HLA-A*0201-binding peptide derived from a novel tumor-associated antigen PBF. The peptide PBF A2.2 was recognized by CD8^+ ^T cells in three of five HLA-A*0201-positive patients with osteosarcoma and induced an oligoclonal CTL line and five CTL clones from these CD8^+ ^T cells. The CTL line, CTL 5A9, and five CTL clones all exhibited specific cytotoxic activity against PBF A2.2-pulsed T2 cells and allogeneic osteosarcoma cell lines expressing both HLA-A*0201 and PBF. In addition, CTL 5A9 lysed autologous osteosarcoma cells derived from fresh biopsy specimens. These findings indicated that PBF A2.2 served as a CTL epitope on osteosarcoma cells in the context of HLA-A*0201.

Interestingly, CTL 5A9 and the five CTL clones lysed an allogeneic osteosarcoma cell line (KIKU) that expressed PBF and HLA-A*0206, but not HLA-A*0201. This suggested that the peptide PBF A2.2 might also be presented on osteosarcoma cells in the context of HLA-A*0206, as seen for other tumor-associated antigens [27,28]. Alternatively, CTL 5A9 and the five CTL clones might cross-react with an allogeneic antigen presented by HLA-A*0206, B*4006, or -Cw*0802, that was not shared by OS2000 and HOS, on KIKU cells. To determine these possibilities, cytotoxicity assays with other target cells that express both PBF and HLA-A*0206 will be required. Thus far, the proof of immunogenicity of PBF has been limited to an HLA-B55-positive patient [12] and HLA-A24-positive patients with osteosarcoma [17]. Our findings in the present study extend the application of PBF-targeting immunotherapy towards patients with HLA-A*0201 and potentially those with HLA-A*0206.

The frequency of the PBF A2.2-specific CTL precursors ranged from 2 × 10^-7 ^to 5 × 10^-6 ^in patients with osteosarcoma. On the other hand, the frequency of the PBF A2.2-specific CTL precursors in healthy donors ranged from 1 × 10^-7 ^and 3 × 10^-7^. In our previous study [17], the frequency of PBF A24.2-specific CTL precursors was between 5 × 10^-7 ^and 7 × 10^-6^. In melanoma patients, the MAGE3.A1-specific CTL precursor frequency was less than 10^-7 ^in normal individuals and non-vaccinated patients as determined by the LD/MLPC/tetramer procedure [29]. Notably the frequency of MAGE3.A1-specific CTL precursors rose to 10^-6 ^following vaccination [29]. Therefore the significance of measuring the frequency of peptide-reactive CTL precursors is to determine the baseline frequency in non-vaccinated patients for forthcoming clinical vaccination trials.

The frequency of CTL precursors is generally under the detection limit of the standard tetramer analysis [30-33] so the LD/MLPC/tetramer procedure was developed. The presence of false-positive wells is a concern in the LD/MLPC/tetramer procedure. To reduce this, we double-stained cells with A2/PBF A2.2 tetramer-PE and control tetramer-FITC (this detects cells that nonspecifically bind tetramers). In tetramer-positive wells, percentages of tetramer-positive cells varied from 0.03% to 0.39% in the present study. The variation of the percentages of tetramer-positive cells conceptually reflects the differing proliferation activities of a single CTL precursors seeded in each well, but does not affect calculation of the frequency of CTL precursors. Therefore, it is critical in the LD/MLPC/tetramer procedure to detect cells that react with the A2/PBF A2.2 tetramer despite the quite low percentages.

## Conclusion

The present study demonstrates the immunogenicity of peptide PBF A2.2 in HLA-A*0201-positive patients with osteosarcoma. The PBF A2.2 peptide is a novel antigenic peptide naturally presented on osteosarcoma cells in the context of HLA-A*0201 and, potentially, HLA-A*0206. This extends the availability of PBF-derived therapeutic peptide vaccines for patients with osteosarcoma.

## Competing interests

The authors declare that they have no competing interests.

## Authors' contributions

TT designed the study, carried out most experiments and drafted the manuscript.

SK made a substantial contribution to critical reading. AT performed single-cell sorting. MM and MK participated in the preparation of patients' samples. SK, TW, MK and SN contributed to collecting patients' samples with the informed consent. SK, TT, TW, TY and NS participated in its design and coordination. All authors read and approved the final manuscript.

## Supplementary Material

Additional file 1 Sequences and binding affinities of PBF-derived peptides with HLA-A*0201 binding motif. *Binding score was determined by BIMAS HLA Peptide Binding Predictions. ^†^The affinity of each peptide (50 μg/ml) was evaluated by a HLA class I stabilization assay. Click here for file

Additional file 2 **Clinical picture and frequency of anti-PBF A2.2 peptide CTLs in PBMC of patients with osteosarcoma**. P: primary tumor, M: metastatic tumor. ^†^Frequency of anti-PBF A2.2 CTLs among CD8+ cells. ^‡^Parentheses indicate that the tumor had been resected at the time of blood sampling. ^§^Magnetically separated CD8+ cells. Irradiated peptide-pulsed CD8- cells were used as stimulator. Click here for file
